# A Critical Review of Risk Assessment Models for *Listeria monocytogenes* in Meat and Meat Products

**DOI:** 10.3390/foods13030359

**Published:** 2024-01-23

**Authors:** Ursula Gonzales-Barron, Vasco Cadavez, Juliana De Oliveira Mota, Laurent Guillier, Moez Sanaa

**Affiliations:** 1 Centro de Investigação de Montanha (CIMO), Instituto Politécnico de Bragança, Campus de Santa Apolónia, 5300-253 Bragança, Portugal; vcadavez@ipb.pt; 2 Laboratório para a Sustentabilidade e Tecnologia em Regiões de Montanha, Instituto Politécnico de Bragança, Campus de Santa Apolónia, 5300-253 Bragança, Portugal; 3 Department of Nutrition and Food Safety, World Health Organization (WHO), CH-1211 Geneva, Switzerland; deju@who.int; 4 Risk Assessment Department, French Agency for Food, Environmental and Occupational Health & Safety (Anses), 14 Rue Pierre et Marie Curie, 94701 Maisons-Alfort, France; laurent.guillier@anses.fr

**Keywords:** systematic review, exposure assessment, simulation, deli meats, sausages, listeriosis

## Abstract

A review of the published quantitative risk assessment (QRA) models of *L. monocytogenes* in meat and meat products was performed, with the objective of appraising the intervention strategies deemed suitable for implementation along the food chain as well as their relative effectiveness. A systematic review retrieved 23 QRA models; most of them (87%) focused on ready-to-eat meat products and the majority (78%) also covered short supply chains (end processing/retail to consumption, or consumption only). The processing-to-table scope was the choice of models for processed meats such as chorizo, bulk-cooked meat, fermented sausage and dry-cured pork, in which the effects of processing were simulated. Sensitivity analysis demonstrated the importance of obtaining accurate estimates for lag time, growth rate and maximum microbial density, in particular when affected by growth inhibitors and lactic acid bacteria. In the case of deli meats, QRA models showed that delicatessen meats sliced at retail were associated with a higher risk of listeriosis than manufacture pre-packed deli meats. Many models converged on the fact that (1) controlling cold storage temperature led to greater reductions in the final risk than decreasing the time to consumption and, furthermore, that (2) lower numbers and less prevalence of *L. monocytogenes* at the end of processing were far more effective than keeping low temperatures and/or short times during retail and/or home storage. Therefore, future listeriosis QRA models for meat products should encompass a processing module in order to assess the intervention strategies that lead to lower numbers and prevalence, such as the use of bio-preservation and novel technologies. Future models should be built upon accurate microbial kinetic parameters, and should realistically represent cross-contamination events along the food chain.

## 1. Introduction

Although invasive listeriosis is a rare disease, it is its high rate of mortality which makes it of significant public health concern worldwide. In 2021, listeriosis occupied the fifth place among the most frequent zoonoses in the European Union (EU), with 2183 confirmed cases in 27 EU Member States and with a high fatality rate of 13.7% [1]. The overall EU trend of listeriosis rates were fairly constant in the period 2017–2020; however, in 2021, the EU notification rate increased by 14% (this is, from 0.43 per 100,000 population in the year 2020, up to 0.49 per 100,000 population in the year 2021). According to the US Centers for Disease Control and Prevention [2], the reported incidence rates of listeriosis in the USA are significantly lower than those of the EU; nonetheless the USA data showed a growing trend between the years 2012/2013 and 2020. During this time span, the crude incidence rates increased from 0.23 to 0.27 cases per 100,000 population.

Reasons for the increase in the incidence of listeriosis could be as follows: (i) the increase in the share of the elderly population in the demographics of industrialised countries [3]; (ii) the greater purchase of convenience/RTE foods due to the limited time available for preparing meals at home [4]; and (iii) the increased consumption of high-risk RTE foods [5]. Consumers have increased preference for “trendy foods” or foods perceived as healthy, whose safety heavily relies on mild treatments, such as plant-based foods (e.g., sprouts, raw quinoa grains), seafood preparations containing raw/macerated ingredients (e.g., gravad fish), etc.

According to the EFSA report [1], the food vehicles implicated in the strong-evidence listeriosis outbreaks in 2021 belonged to the categories “Fish and fish products” (four outbreaks), “Broiler meat and products thereof” (one outbreak), and “Other mixed red meat and products thereof’ (one outbreak). Thus, meat products and seafood continue to be important food vehicles, as they were in the decade 2010–2020, with a pooled share of 30.2% and 22.6%, respectively, of the total strong-evidence listeriosis outbreaks in the EU.

In this context, many quantitative risk assessment (QRA) models have focused on meat products—as important vehicles of transmission—to estimate the risk and incidence of invasive listeriosis linked to their consumption. The present study aims (i) to perform a critical review of the QRA models currently published for listeriosis linked to the consumption of meat and meat products; (ii) to discuss the relative effectiveness of the risk mitigation measures evaluated in the various QRA models as what-if scenarios; and (iii) to extract key messages and suggestions for future QRA models. 

## 2. Materials and Methods

QRA models were retrieved through a literature search on Scopus and PubMed^®^ published between 1 January 1998 and 18 May 2022 (date of the searches). The searches in title, keywords and abstract were carried out using logically connected terms ((“risk assessment” OR exposure OR quantitative microbial OR risk modelling OR modeling OR simulation* OR second-order OR “second order” OR “risk management”) AND (“*L. monocytogenes*” OR “*Listeria monocytogenes*” OR listeriosis)) properly arranged in the syntaxes of the literature search engines. The full systematic review and information extraction processes are described in Gonzales-Barron et al. [6]. QRA models conducted in any region of the world were included. The present review focuses only on meat products, which were the subject of 25 studies [7,8,9,10,11,12,13,14,15,16,17,18,19,20,21,22,23,24,25,26,27,28,29,30,31].

## 3. Results

### Description of Collected QRA Models

Table 1 summarises the main features of the 24 QRA models of *Listeria monocytogenes* retrieved for meat or meat products, while Table 2 compiles the predictive microbiology models and summarised results from what-if scenarios and sensitivity analysis, excerpted from the models. Most of the models developed in the American continent represented the food production conditions of the USA (9). Other 4 models were developed in Argentina (2), Canada (1) and Chile (1). Five QRA models investigated the risk of listeriosis in Europe (Italy, Greece, Spain, France and EU), whereas the other three investigated in Asia (one from China and two from Malaysia). One QRA model pertained to the risk of listeriosis in the Australian population, whereas two models were not linked to any specific geographical location (Table 1). 

Only three models investigated the risk of listeriosis from non-RTE foods; namely, poultry and beef [22] and chicken offal [30,31]. The other 21 models focused on RTE processed meats, of which 12 were explicitly deli meats. The remaining eight pertained to fermented sausages (×3: [8,10,18]), bulk-cooked meat [9], dry-cured pork shoulder [10], miscellaneous processed meats [17], and packaged heat-treated meats [25,27]. 

From the 12 deli meats models, four of them [7,14,20,28] compared the risk of listeriosis between manufacture pre-packed and retail-sliced deli meats (Table 1).

The majority of the listeriosis QRA models for meat products considered contamination pathways from end of processing or retail to table (15/23), with 10 of them investigating deli meats. QRA models of longer scope (processing-to-table) were reserved mostly for processed meats rather than deli meats (five models), namely, high-pressure-processed chorizo, bulk-cooked meat, fermented sausage and dry-cured pork shoulder, in which the effects of processing stages were characterised. Only one QRA model for deli meats covered the processing-to-table scope [7]. Three models consisted only of the consumption module, from which one pertained to deli meats [29]. No single QRA model included a primary production module (Table 1).

Processed meat products may be contaminated with *L. monocytogenes* at different stages: either raw materials are contaminated and processing stages are unable to reduce pathogen’s populations; or contact with contaminated raw materials contaminates surfaces or operators, which may take place at any phase between the meat processing plant and the consumer’s home. For such reasons, nearly half of the QRA models (11/23) attempted to characterise cross-contamination, with modules placed during food processing [7,8], at retail [7,9,15,16,23,26,28] and during handling at home [22,24,29,31] (Table 1). Except for Pouillot et al. [23], who developed a rather complex discrete event approach to model cross-contamination at retail, simple transfer coefficients were applied in the QRA models to depict cross-contamination during processing (i.e., from slicing machine), at retail (i.e., from slicing machines and from other environmental elements) and at home (i.e., from fridge, hands and to cooked meat).

The QRA models retrieved varied in degree of architectural complexity. For the estimation of the exposure or the risk, only 18% of the models established a separation between variability and uncertainty [9,14,15,16,17]. All QRA models, except two [30,31], employed predictive microbiology models, including microbial growth, survival and competition models (Table 2). The lag phase duration of *L. monocytogenes* was not represented in 14 models [8,9,17,18,19,20,21,22,23,26,27,29,30,31], whereas only 3 QRA models [23,25,28] employed time–temperature trajectories to more realistically estimate the growth of *L. monocytogenes* along the storage process (Table 1).

Five QRA models considered mortality, apart from illness, as endpoint for risk estimation, having in common that all of them used the dose-response model from FDA-FSIS [11,14,20,21,28]. In one model, no risk estimation was carried out, since authors were interested in modelling only exposure assessment [8]. The exponential “single-hit” dose function was the choice for all models, of which the most common approach was that of FAO/WHO [18] (10 models), followed by FDA-FSIS [17] (8 QRA models), Pouillot et al. [32] (5 QRA models), Lindqvist and Westoo [35] (2 QRA models) and Pouillot et al. [34]. EFSA BIOHAZ [27] proposed a dose–response model based on that of Pouillot et al. [32] but stratified by gender and age classes instead of by consumers’ pre-conditions.

Most of the QRA models assessed the impact of what-if scenarios, which can be understood as risk factors or intervention strategies (20 models); sensitivity analysis on *L. monocytogenes* counts, on dose per serving or on listeriosis risk as response variables was performed in 9 models (Table 2).

## 4. Discussion

### 4.1. Risk Factors and Control Measures Assessed at Processing

According to the systematic review conducted by Gonzales-Barron et al. [6], out of 65 QRA listeriosis QRA models retrieved since 1998, most of them focused on meat and meat products (35.4%)—followed by dairy products (27.7%). The scope of the listeriosis QRA models linked to meats begins upstream in the supply chain, as processing or end-of-processing/retail, and not earlier, despite farms and slaughtering being important sources of contamination of meats and meat products [37,38,39,40]. This may happen because many tracking survey studies have shown that the contamination of the final meat products commonly occurs in the slaughterhouse or at a retail level, and rarely directly from food-producing animals (i.e., faecal contamination). As early as in 1996, Nesbakken et al. [41] concluded that post-slaughtering processing was a significant source of contamination, and that it was in the cutting room environment where contamination is amplified. Later, Kathariou [42] was more conclusive, stating that, in general, the primary source of contamination before handover to consumers was the processing stage. Thévenot et al. [43] detected both persistent and sporadic strains of *L. monocytogenes* in meat processing environments, with a high genotype diversity. Therefore, even if *L. monocytogenes* enters the processing plant at low levels, some strains may survive in biofilms, persist in the processing environment and contribute to both environment and RTE meat product contamination and recontamination.

For RTE meat products, the most frequently applied hurdles are the use/application of growth-inhibitors (GI) (i.e., nitrites, lactate, diacetate, etc.), biopreservatives (i.e., lactic acid bacteria (LAB) cultures), post-process lethality treatments (i.e., in-package pasteurisation, irradiation, high-pressure processing, etc.), vacuum-packaging and cold storage. The effectiveness of a post-process lethality treatment, the use of GI and sampling testing at the end of processing was evaluated in the only QRA model for deli meats covering processing to retail [7]. This research found that the combination of the two hurdles—post-process lethality treatment (pasteurisation or UV) and the application of GI packaging (1.5–3.0% lactate alone or in combination with 0.125–0.25% diacetate)—was more effective in reducing the annual cases of listeriosis (92.5% reduction) than when used alone (38% or 80% reduction, respectively), and far more effective than implementing a verification sampling of 60 samples per month in small, medium and large facilities (15% reduction) (Table 2). Such scenarios were tested conforming to FSIS [44] rules to effectively control *L. monocytogenes* in RTE foods.

Other hurdles applied to meat products different than deli meats were evaluated in processing-to-retail QRA models, such as the application of high-pressure processing (HPP) in chorizo [8], the addition of LAB culture in fermented sausage [10] and the effect of lower water activity during ripening (longer dehydration) in fermented sausage and dry-cured pork shoulder [10]. Possas et al. [8] demonstrated that HPP was an efficient intervention to reduce the prevalence of contaminated chorizo packs, although it depended on the application time. HPP at 600 MPa for 3, 6 or 9 min reduced the mean prevalence of *L. monocytogenes* in contaminated chorizo packs at consumption by 90, 97 or >99.9%, respectively, when initial contamination in the batter was below 100 CFU/g. Nonetheless, they showed that, if 150 ppm nitrites were removed from the chorizo formulation, and HPP was applied at 600 MPa for 6 min or 9 min, the resulting mean prevalence of contaminated chorizo packs would increase by 66% (from 0.09% to 0.15%) or 100% (from 0.01% to 0.02%), respectively, in comparison to those scenarios where HPP was applied and nitrites were kept. Other scenarios relative to alterations of processes were evaluated by Brusa et al. [10]. They estimated that, when a certain LAB cocktail is added to the fermented sausage formulation, the final concentration of *L. monocytogenes* was lower than 100 CFU/g in 98.2% of the sausages, as opposed to the 73.7% attained when LAB was not added. They also predicted a relationship between the final pH of the sausage and the odds ratios of the risk of listeriosis. As the final pH decreased from 5.9 to 5.7, 5.5 and 5.3, the odds of acquiring listeriosis diminished from 2.52 to 1.97, 1.61 and 1.03 times higher than the odds of acquiring listeriosis from sausages of pH = 5.1. Greater dehydration was also an effective hurdle; decreasing the a_w_ of fermented sausages during ripening to ≤0.92 also reduced the risk of listeriosis in a magnitude 1.7 times lower than sausages with a_w_ ≥ 0.93, whereas decreasing the a_w_ of dry-cured pork shoulder by salting to values ≤ 0.93 reduced the risk of listeriosis to a greater extent (27 times lower). In the sensitivity analyses for both fermented sausage and dry-cured pork shoulder, the prevalence of *L. monocytogenes* in raw meat was associated with the risk of listeriosis (r = 0.28 and 0.13, respectively); however, whereas, in the fermented sausage, variables related to pH drop (i.e., the use of LAB, fermentation temperature, pH reached during fermentation, LAB concentration in the fermented sausage) were more important than the water activity of the final sausage, for the dry-cured pork shoulder, the water activity during salting was the most correlated with listeriosis risk.

Six of the QRA models [11,14,15,16,20,21,24] assessed the impact of GIs in deli meats, more specifically the combined application of lactate and diacetate, which have been long recognised as capable of suppressing pathogenic growth in foods with neutral pH. Their effectiveness in reducing listeriosis risk has been shown to be variable. The FSIS QRA model [21] estimated that the formulation of RTE meat and poultry deli meats with GI would reduce the mean annual death cases by 78% in the elderly; Endrikat et al. [20] predicted that the use of GI in pre-packed deli meats and in retail-sliced deli meats would reduce the mean annual deaths by 55% and 82%, respectively, in the elderly population. Pradhan et al. [11] stated that products formulated with GIs would decrease the mean annual deaths in the elderly 7.8-, 3.7- and 2.5-fold for RTE turkey, roast beef and ham, respectively, whereas Falk et al. [24] estimated greater reductions in the median cases of listeriosis, namely, 78-fold for ham delicatessen meat, 56-fold for beef delicatessen meat and 49-fold for hotdog, when they were formulated with GIs. Gallagher et al. [15,16] estimated that, if all RTE products sold in delis (RTE turkey, ham and roast beef) were reformulated with GI, the mean risk of listeriosis would be reduced by 95.2% in the susceptible population.

Many QRA models demonstrated, through what-if scenarios, how greater risk reductions can be attained by reducing the initial prevalence and/or counts of *L. monocytogenes* in foods at the end of processing or retail [12,13,17,24,25,29]. In FDA-FSIS [17], higher reduction levels, rather than decreasing storage time and storage temperature, were obtained by reducing the initial concentration of *L. monocytogenes* at the start of retail in processed meats, which they hypothesised could be achieved through a processing-level lethal intervention. This QRA model estimated that reducing the initial mean contamination by 1.0 log would reduce the number of deaths in the elderly population by 50%, whereas reducing contamination in 2.0 logs would result in a 74% reduction. Many years later, in the QRA model built by Pérez-Rodríguez et al. [25], a comparable level of reduction in listeriosis cases (89% reduction) was obtained when the maximum initial concentration of *L. monocytogenes* in packaged, heat-treated meat products was reduced in 2.0 logs. Such a reduction rate was greater than those of the scenarios of decreasing the storage temperature and time to consumption. Ross et al. [12,13], simulating what would be the application of a milder listericidal treatment during processing that would achieve 1–2 log mean reduction in *L. monocytogenes*, calculated a 150-fold decrease in the mean predicted annual listeriosis from luncheon meats, cooked sausages and pâtés. Simulating a greater reduction in *L. monocytogenes* initial counts of 3–4 logs, they estimated a ~600-fold decrease in the annual cases of listeriosis. Comparable high levels of risk reduction were found by Falk et al. [24]—952-, 279-, 381- and 116-fold decreases in the median cases of listeriosis for turkey deli meat, ham delicatessen meat, beef delicatessen meat and hot-dog, respectively—if the initial mean concentration of *L. monocytogenes* at retail would be reduced from 75 CFU/g to 1 CFU/g. Furthermore, Yang et al. [29] also corroborated that the initial contamination level at retail (r = 0.29) was the main driver of mortality in the intermediate-age population due to the consumption of vacuum-packed and freshly sliced deli meats, a much stronger factor than storage temperature (r = 0.09) and storage time (r = 0.03) (Table 2).

### 4.2. Risk Factors and Control Measures at Retail and Home

Reducing storage temperature had a greater effect on reducing the risk of listeriosis than reducing storage time. In Pradhan et al. [14], the scenario of reducing the maximum storage temperature to 7 °C reduced the median number of deaths by 64% and 80% for pre-packaged ham elaborated without and with GIs, respectively, and by 62% and 79% for retail-sliced ham elaborated without and with GIs, respectively. In contrast, when the mean storage time was decreased from 28 to 16 days, the median numbers of deaths for the above products were reduced, to a lesser extent, by 24%, 51%, 32% and 57%, respectively. 

Accordingly, these authors estimated that the concentration of *L. monocytogenes* at the end of retail was mainly driven by storage temperature (r = 0.65), followed by the lag time (r = −0.49) and the storage time (r = 0.33). A sensitivity analysis on listeriosis cases performed by Falk et al. [24] also showed that the listeriosis cases from deli meats was more affected by the consumer’s refrigerator temperature than by storage time (r not provided), whereas, in Yang et al. [29], the consumers’ refrigeration temperature (r = 0.09) and the refrigeration time (r = 0.03) were both poorly correlated with mortality in the intermediate-age population.

In a QRA model on deli meats, at the stage of retail, Gallagher et al. [15,16] showed that, during retail, setting the deli temperature no higher than 5 °C would reduce the mean risk of listeriosis in the susceptible population by 16.3%, whereas shortening the time in retail delis from 7 to 4 days would have no effect on the mean risk of listeriosis. Likewise, at the consumer level, keeping home refrigerators at temperatures lower than 5 °C would reduce the risk by 99.99%, whereas consuming the deli meats within 4 days after purchase would reduce the mean risk by 99.0%. On the other hand, interestingly, there were two QRA models [25,26] where decreasing the storage temperature produced a similar effect on the final risk as reducing the storage time. In Pérez-Rodríguez et al. [25], a decrease of 1–2 °C in the dynamic temperature profiles, and a decrease in the time to consumption of 25%, led both to a 37–38% reduction in the cases of listeriosis per million servings of packaged heat-treated meat products, whereas, in Tsaloumi et al. [26], setting a use-by date of 14 days from the time of slicing, or reducing consumers’ storage temperature from a mean of 6 °C to 5 °C, would reduce the median cases of listeriosis from seven (baseline scenario of no use-by date and mean of 6 °C storage) to zero. Duret et al. [28] explored the link between the cold chain for cooked ham (including transport, supermarket cold storage, display cabinets, consumer transport and home refrigerator) and the associated listeriosis risk, together with the food wasted due to spoilage bacteria (LAB) and the cold-chain electricity consumption. A set of eight intervention strategies was tested to assess their effect on the three criteria investigated: food safety, food waste and energy consumption. The results showed that changing the thermostat of the home refrigerator has a high effect on listeriosis risk and food waste, but a limited effect on electricity consumption. Conversely, changing the airflow rate in the cabinet has a significant effect on electricity consumption but a negligible impact on listeriosis risk and food waste.

### 4.3. Cross-Contamination

Multiple studies have shown that delicatessen meats sliced in retail tend to have a higher level of bacterial contamination than deli meats sliced in factories [21,45,46]. The QRA model for pre-packed and retail-sliced deli meats from Endrikat et al. [20] estimated a relative risk of deli meats sliced at retail versus sliced in factories of 4.89 in the elderly population. A model published by FSIS [21] also found a similar result: retail-sliced deli meats presented annual death cases 80% higher than pre-packed deli meats. Both studies showed, by means of regression trees analysis, that the most important determinant of risk, after age of consumers, was slicing location (whether retail-sliced or pre-packed) and, according to them, this was more determinant than the presence of GI. Likewise, Pradhan et al. [14] demonstrated that the malpractice of storing in home refrigerators at temperatures higher than 10 °C accounts for ~17% and 32% of the predicted deaths associated with pre-packaged ham without and with Gis, respectively, whereas higher estimates of ~20% and 41% of the deaths are associated with retail-sliced ham without Gis and with Gis, respectively (Table 2).

These recurrent findings have suggested that the preparation of such RTE foods in retail shops augments the risk of contamination. In the retail environment, there are sources of contamination or cross-contamination, routes of spreading of *L. monocytogenes* and potential problems with hygiene monitoring. Pouillot et al. [23] developed a discrete-event modelling framework of cross-contamination from object to object, from food to object and from object to food; they assumed the presence of *L. monocytogenes* niches or harbourage sites allowing the release, at a given frequency, of a given number of cells to a site (i.e., utensils, slicers, food contact surfaces, scales, sinks, handles, floors, etc.). The model demonstrated that, (1) when more *L. monocytogenes* cells enter the retail environment, the risk is increased, regardless of the origin of the cells (from niches in the retail environment or from cells on incoming RTE food from the manufacturer) and that (2) a high frequency of cross-contamination events (daily versus weekly) has more impact than a high concentration of *L. monocytogenes* per cross-contamination event (100 versus 1000 CFU). Pouillot et al. [23] predicted that the risk of products from retail shops, with highly contaminated incoming RTE food that supports *L. monocytogenes* growth, is six times higher than the risk from shops that have an equally highly contaminated incoming RTE food, yet one that does not support growth. This means that most of the increase in the risk of products from these deli shops arises from cross-contamination to RTE foods supporting the growth of *L. monocytogenes*. Therefore, both the elimination of *L. monocytogenes* niches in retail deli establishments and efficient temperature control are crucial in attaining a lower risk of listeriosis. Yang et al. [29] indicated that cross-contamination at home has a lower contribution to listeriosis risk than the contamination levels that can be attained during retail.

### 4.4. L. monocytogenes Lag Phase as a Driver of Risk Estimation

Finally, some QRA models have assessed the impact of the lag phase duration of *L. monocytogenes* on the final risk. The lag phase assumption is of particular importance in the exposure assessments of foods, such as meat products, that are formulated with compounds or additives that act by extending the lag phase duration of microorganisms. In their QRA model for deli meats, Pradhan et al. [11] illustrated the importance of including the lag phase duration, by indicating that the mean numbers of deaths and illnesses for ham and roast beef formulated without Gis were 2.4- and 1.9-fold lower when lag phase was considered, than those obtained without lag phase (long mean lag phases of 5.9 and 5.1 days assumed for ham and roast beef without GI, respectively). Later, the same authors [14] estimated that lag time was the second most important variable (r = −0.49) driving *L. monocytogenes* concentration at the end of retail, just after retail storage temperature. Likewise, whereas, in the QRA model of Falk et al. [24] for delicatessen meats and hot dogs, the lag time duration was a moderate driver (r = −0.13 to −0.27) of the number of listeriosis cases, Pérez-Rodríguez et al. [25] quantified a reduction of 57% in the number of cases of listeriosis per million servings if the lag time was included in the QRA model for packaged heat-treated meat products. Conversely, using a sensitivity analysis, Duret et al. [34] showed that the latency modeling approach (population versus individual) was not an important parameter for exposure. The authors chose the simplest approach for estimating risk [28]. 

### 4.5. Model Availability

Sharing risk assessment models is crucial to ensure the transparency of the methodology and facilitate reusability. This holds particular significance in the realm of scientific research, where there is a growing emphasis on reproducibility and open science [47]. By sharing these models, researchers enable others to scrutinise their work, detect potential biases and use the models for their own datasets. Beyond transparency, the sharing of models streamlines reusability, a boon for researchers who may lack the resources to develop their own models [48].

Among the studies published, two grant access to scripts or spreadsheets [25,27], while two propose sharing the utilised models upon request [23,28] (details regarding model-sharing characteristics are available in the Supplementary Material of this article). For the other models, there is no indication of their availability. Several studies refer to a site that is no longer maintained [11,12,17], highlighting the challenge of reproducibility over time. As software evolves and resources vanish, such as the need for website maintenance, reproducing calculations becomes increasingly challenging [49].

## 5. Conclusions

None of the 23 QRA models retrieved simulated primary production or slaughtering; their scope mostly focused on end-of-processing or retail-to-consumption, although they indirectly evaluated the impact of growth inhibitor and lethal treatments on the final risk estimate. Most of the QRA models were carried out for RTE meat products such as deli meats, since these products support the growth of *L. monocytogenes*, have a long shelf-life and are very susceptible to cross-contamination in the processing and retail environments. The outputs of the QRA models agreed that deli meats sliced at retail sites lead to higher risks of listeriosis than manufacture pre-packed deli meats. Cross-contamination events are represented in approximately half the QRA models retrieved. However, cross-contamination modelling should not be overlooked, since *L. monocytogenes* has been frequently detected in processing and retail environments, revealing that persistent strains could be isolated from contact surfaces even after cleaning and disinfection, and even with recovery times of up to three years in the meat processing environment. QRA models widely agreed on the fact that controlling (reducing) the initial concentration of *L. monocytogenes* at the end of processing—which could be achieved through growth inhibitors or through the application of heat treatment or high-pressure processing—would be far more effective than keeping storage temperatures low or reducing storage times. If a meat product contains a growth inhibitor compound or a lactic acid bacteria culture starter is added, it is important to determine how these preservatives would affect the lag time duration and maximum population density of *L. monocytogenes*, because QRA models have demonstrated the moderate impact that those two parameters exert on the final risk estimate. Future QRA models should include cross-contamination modules along the food chain, should be based on accurate microbial kinetic parameters and should represent the effects of new technologies and/or intervention strategies, such as high pressure processing, functional starter cultures, bacteriocins and bioactive packaging. Models should also allow the assessment of the impact of effective cleaning and sanitation programmes in processing plants, as well as the impact of end-product batch microbiological testing.

## Figures and Tables

**Table 1:** Characteristics of quantitative risk assessment models of *L. monocytogenes* from consumption of meat or meat products by scope.

**Scope**	Food	RTE	Cross-Conta-Mination	DR—End-Point	Type of DR Model	DR Sub-Populations	Strain Variability	Temp Profiles/ Lag Time	Country	Source
**Processing-to-table**	Deli meats: turkey, ham and beef—pre-packed and retail-sliced	Yes	Yes: Processing: a transfer coefficient modelled; and for retail-sliced meats, cross-contamination was modelled at retail	Exp—I	FAO/WHO [18]	High-risk/Low-risk	Variability of strains implicit in r	No/Yes	USA	Tang [7]
	HPP-treated chorizo	Yes	Yes: Processing—slicing—transfer coefficient	NA	NA	NA	No strain variability considered	No/No	Spain	Possas et al. [8]
	Bulk-cooked meat	Yes	Yes: Retail: from other products and from the retail environment	Exp—I	FAO/WHO [18]	High-risk/Low-risk	Variability of strains implicit in r	No/No	China	Sun et al. [9]
	Fermented sausage	Yes	No	Exp—I	Pouillot et al. [32]	Multiple	Virulence and host susceptibility explicit in r distribution	No/Yes	Argentina	Brusa et al. [10]
	Dry-cured pork shoulder	Yes	No	Exp—I	Pouillot et al. [32]	Multiple	Virulence and host susceptibility explicit in r distribution	No/Yes	Argentina	Brusa et al. [10]
**End Process-to-table**	Deli meats: ham, turkey and roasted beef	Yes	No	Mouse-Epi—I, D	FDA-FSIS [17]	Multiple	Virulence of different strains represented in DR	No/Yes	USA	Pradhan et al. [11]
	Luncheon meats, cooked sausages, pâtés	Yes	No	Exp—I	FAO/WHO [18]	High-risk/Low-risk	Variability of strains implicit in r; Some strain variability in LM growth rate	No/Yes	Australia	Ross et al. [12,13]
	Manufacture- (pre-packaged) and retai—sliced ham and turkey	Yes	No	Mouse-Epi—D	FDA-FSIS [17]	High-risk	Virulence of different strains represented in DR	No/Yes	USA	Pradhan et al. [14]
	Deli meats: RTE turkey, ham and roast beef	Yes	Yes: Retail	Exp—I	FAO/WHO [18]	High-risk/Low-risk	Variability of strains implicit in r	No/Yes	USA	Gallagher et al. [15,16]
**Retail-to-table**	Processed meats: Frankfurters, fermented sausages, deli meats, pâté	Yes	No	Mouse-Epi—I	FDA-FSIS [17]	Multiple	Virulence of different strains represented in DR	No/No	USA	FDA-FSIS [17]
	Fermented meat	Yes	No	Exp—I	FAO/WHO [18]	High-risk/Low-risk	Variability of strains implicit in r	No/No	Non-specific	FAO-WHO [18]
	Hams	Yes	No	Exp—I	FAO/WHO [33]	Multiple	NA	No/No	Italy	Giovaninni et al. [19]
	Pre-packed deli meats/retail-sliced deli meats	Yes	No	Mouse-Epi—I, D	FDA-FSIS [17]	Multiple	Virulence of different strains represented in DR	No/No	USA	Endrikat et al. [20]
	RTE meat and poultry deli meat	Yes	No	Mouse-Epi—I, D	FDA-FSIS [17]	Multiple	Virulence of different strains represented in DR	No/No	USA	FSIS [21]
	Poultry and beef	No	Yes: Handling at home	Exp—I	FAO/WHO [18]	General	Variability of strains implicit in r	No/No	Chile	Foerster et al. [22]
	Retail delicatessens	Yes	Yes: Retail: series of events when serving deli meat	Exp—I	FAO/WHO [18]	High-risk/Low-risk	Variability of strains implicit in r	Yes/No	USA	Pouillot et al. [23]
	Delicatessen meats/hotdogs	Yes	Yes: Handling at home	Exp—I	Pouillot et al. [32]	Multiple	Virulence and host susceptibility explicit in r distribution	No/Yes	Canada	Falk et al. [24]
	Packaged heat-treated meat products (cooked meat, sausage, pâté)	Yes	No	Exp—I	Pouillot et al. [32]	Multiple	Challenge test data from a mixture of strains; Virulence and host susceptibility explicit in r distribution; h0 distribution of variability in physiological state of cells; LM DR modelling explicitly considers variability in strain virulence and in susceptibility across populations	Yes/Yes	Non-specific	Pérez-Rodríguez et al. [25]
	Retail-sliced cooked meats	Yes	Yes: Retail: from slicing machine	Exp—I	Pouillot et al. [32]	High-risk/Low-risk	NA	No/No	Greece	Tsaloumi et al. [26]
	RTE cooked meat, RTE sausage, patê	Yes	No	Exp—I	EFSA BIOHAZ [11] based on Pouillot et al. [32]	Multiple (sex/age group)	Mixture of strains used in challenge test; Virulence and host susceptibility explicit in r distribution	No/No	Non-specific	EFSA BIOHAZ [27]
	RTE cooked meat	Yes	No	Exp—I	Pouillot et al. [34]	Multiple	NA	Yes/Yes	France	Duret et al. [28]
**Consumption**	Vacuum-packed and freshly sliced deli meats	Yes	Yes: Handling at home: transfer rates from refrigerator and from hands	Mouse-Epi—D	FDA-FSIS [17]	Intermediate-age population	Virulence of different strains represented in DR	No/No	USA	Yang et al. [29]
	Cooked chicken offal	No	No	Exp—I	FAO/WHO [18]; Lindqvist and Westöö [35]; FDA-FSIS [17]	Multiple	Strain diversity implicit in r	No/No	Malaysia	Kuan et al. [30]
	Cooked chicken offal	No	Yes: Handling at home: transfer rate to cooked samples	Exp—I	FAO/WHO [18]; Lindqvist and Westöö [35]; FDA-FSIS [17]	Multiple	Strain diversity implicit in r	No/No	Malaysia	Wai et al. [31]

DR: dose–response; Exp: exponential model; Mouse-Epi: Mouse-Epidemiological model; I: illness endpoint; D: death endpoint; NA: not available.

**Table 2:** Microbial kinetic models and main results related to scenarios and sensitivity analysis from quantitative risk assessment models of listeriosis acquired from consumption of meat or meat products.

Scope	Food	Predictive Microbiology Models	What-If Scenarios	Sensitivity Analysis	Model Complexity	Source
**Processing-to-table**	Deli meats: turkey, ham and beef—pre-packed and retail-sliced	Growth (linear, square root for EGR5)	(1) Maximum sampling frequency of 60 samples per month in small, medium and large facilities that produce two lots per day (60–60–60), reduces listeriosis cases by 15% in comparison to the no-testing baseline; (2) Sampling every lot in small, medium and large facilities (60–60–60L) reduces listeriosis cases by 12%; (3) Implementing 100% post-processing lethality (pasteurisation, UV) reduces 38% of the listeriosis cases; (4) Implementing 100% growth inhibiting packaging (1.5–3% lactate alone or in combination with 0.125–0.25% diacetate) reduces cases by 80%; (5) Post-processing lethality and growth inhibiting packaging reduces cases by 92.5%.	ND	Low	Tang [7]
	HPP-treated chorizo	For HPP processing (inactivation of LM as a function of a_w_, pressure intensities and pressure holding time); Storage, retail, transport (survival: biphasic model, secondary model, survival rate as function of temperature)	(1) When no high-pressure processing (HPP) is applied, if LM in pork meat batter is below 1 log CFU/g, the prevalence of contaminated sausage packs at consumption is 3%; (2) When no HPP is applied, 2 log CFU/g LM in pork meat batter increases prevalence in sausage packs at consumption to 10%; (3) HPP at 600 MPa for 3 min reduces the prevalence of contaminated sausage packs at consumption by 90%; (4) HPP at 600 MPa for 6 min reduces prevalence of contaminated sausage packs by 97%; (5) HPP at 600 MPa for 9 min reduces prevalence of contaminated sausage packs by >99.9%; (6) If nitrites 150 ppm is eliminated (0 ppm) and HPP is applied at 600 MPa for 6 min, the prevalence of contaminated sausage packs increases by 66% (from 0.09% to 0.15%); (7) If nitrites 150 ppm is eliminated (0 ppm) and HPP is applied at 600 MPa for 9 min, the prevalence in sausage packs increases by 100% (from 0.01% to 0.02%).	ND	Low	Possas et al. [8]
	Bulk-cooked meat	Growth (linear, square root for EGR5)	(1) If LM at retail had a mean of −2.7 log CFU/g (instead of −1.4 log in the baseline), the final concentration at consumption would be 0 log CFU/g (instead of 0.20 log CFU/g; (2) If cooked meat was stored under unfavourable conditions that causes LM to go >−0.52 log CFU/g at retail, the final mean concentration at consumption would be 1.5 log CFU/g.	ND	Low	Sun et al. [9]
	Fermented sausage	Growth (both growth probability and GR as functions of temp, a_w_, pH, LAC and LAB_cpd)	(1) When LAB culture was added, the final LM concentration was <100 CFU/g in 98.2% of the portions (as opposed to 73.7% when LAB was not added); (2) Higher values of final pH during fermentation led to higher risk of listeriosis. At pH values of 5.3, 5.5, 5.7 or 5.9, the calculated odds ratios were 1.03, 1.61, 1.97 or 2.52 times higher compared with pH < 5.1; (3) When a_w_ was ≤0.92 during ripening, the risk of listeriosis was 1.73 times lower than with a_w_ ≥ 0.93. For increasing a_w_ (0.94, 0.95 and 0.96), the risk of listeriosis was 39, 56 and 60 times greater compared with a_w_ ≤ 0.92.	**Output—risk of listeriosis**: (1) Use of LAB (r = −0.51), (2) Prevalence of LM in raw meat (r = 0.28), (3) Fermentation temperature (r = 0.24), (4) pH reached during fermentation (r = 0.24), (5) LAB counts in the fermented sausage (r = −0.16), (6) a_w_ of sausage (r = 0.13)	Low	Brusa et al. [10]
	Dry-cured pork shoulder	Growth (both growth probability and GR as functions of temp, a_w_, pH, LAC and LAB_cpd)	(1) At a_w_ ≤ 0.93, the listeriosis risk was 27 times lower compared with the product with a_w_ not reduced in the process.	**Output—risk of listeriosis**: (1) a_w_ reached during salting (r = 0.18), (2) Prevalence of LM in raw meat (r = 0.13), (3) Counts of LM in raw meat (r = 0.10), (4) Temperature at which salting was carried out (r = 0.02), (5) pH during salting (r = −0.01)	Low	Brusa et al. [10]
**End Process-to-table**	Deli meats: ham, turkey and roasted beef	Growth (linear model for growth; secondary models for EGR_5 °C, LPD_5 °C as square root models)	(1) The mean deaths and illnesses for ham and roast beef elaborated without GIs were 2.4- and 1.9-fold lower when lag phase was considered than those obtained without lag phase (mean lag phases of 5.9 and 5.1 days for ham and roast beef without GI); (2) If RTE products were formulated with GIs, the mean deaths in the elderly populations would reduce by factors of 7.8, 3.7 and 2.5 for RTE turkey, roast beef and ham, respectively.	ND	Low	Pradhan et al. [11]
	Luncheon meats, cooked sausages, pâtés	Growth (competiion growth model for LM and spoilage bacteria)	(1) A 90% reduction in LM prevalence leads to an 80% risk reduction. Similarly, a 67% reduction in prevalence leads to a 50% reduction in the risk; (2) Any treatment that reduces LM growth rate by 50% reduces the risk by 80–90%; (3) A 3- to 4-log reduction in initial LM concentration (i.e., heat treatment or HPP in the packaged product) results in a ~600-fold reduction in the annual listeriosis cases; (4) A milder post-processing listericidal treatment, assumed to reach a 1–2-log reduction, results in a 150-fold decrease in the annual listeriosis cases.	ND	Low	Ross et al. [12,13]
	Manufacture (pre-packaged) and retail-sliced ham and turkey	NS	(1) Home storage at temperatures >10 °C causes 17 and 32% of the estimated deaths linked to pre-packed ham without and with GIs, respectively, and 20 and 41% of the deaths associated with retail-sliced ham without and with GIs, respectively; (2) If the maximum temperature was limited to 7 °C, the median numbers of deaths would be reduced by 64% and 80% for pre-packed ham elaborated without and with GIs, respectively. The median numbers of deaths would be reduced by 62% and 79% for retail-sliced ham elaborated without and with GIs, respectively; (3) When the mean storage time was reduced from 28 to 16 days, the median numbers of deaths were reduced by 24%, 51%, 32% and 57% for pre-packed ham elaborated without and with GIs, and retail-sliced ham elaborated without and with GIs, respectively; (4) Limiting storage temperature and time to 10 °C and 16 days reduced the annual number of deaths by ~50% for pre-packaged and retail-sliced ham elaborated without GIs; (5) For products elaborated with GIs, the same combination reduced the annual number of deaths by ~75 and 90% for pre-packed and retail-sliced ham, respectively.	**PRODUCTION TO RETAIL PHASE:** **Output—LM at the end of retail**: Storage temperature had the strongest influence on LM growth (r = 0.65), followed by lag time at reference temperature (r = −0.49), storage time (r = 0.33), and growth rate at reference temperature (r = 0.24)	Low: Linked to model of Pradhan et al. (2009)	Pradhan et al. [14]
				**RETAIL PHASE TO CONSUMPTION** **Output—annual deaths in the elderly population**: (1) If maximum storage temperature between retail and consumption is reduced by 2 °C (baseline 21 °C), mean deaths are reduced from 13.5 to 6.5, (2) If maximum storage time between retail and consumption is reduced by 4 days (baseline 45 days), the mean deaths are reduced from 13.5 to 10, (3) If EGR 5 °C is reduced by 0.03 log/day; mean deaths are reduced from 13.5 to 11.5.		
	Deli meats: RTE turkey, ham and roast beef	Growth (linear model, EGR_5 °C for temperature)	(1) Reformulating all RTE products sold in delis with GIs reduces the mean listeriosis risk in the susceptible population by 95.2%; (2) Setting retail deli temperatures no higher than 5 °C reduces mean risk of listeriosis by 16.3%; (3) Shortening time in retail delis from 7 to 4 days has no effect on the mean risk of listeriosis; (4) A decrease in LM concentration on incoming RTE products by a factor of 2 would decrease the listeriosis risk of RTE foods prepared at retail by 10 to 24%; (5) Keeping all home refrigerators at temperatures <5 °C reduces mean risk by 99.8%; (6) Consuming all products within up to 3–4 days reduces mean risk by 99.0%.	ND	Medium: Different products considered with and without GI.	Gallagher et al. [15,16]
**Retail-to-table**	Processed meats: Frankfurters, fermented sausages, deli meats, pâté	Growth (linear model, square root model for EGR)	(1) Reducing the home storage time for deli meats from 28 days (baseline) to 14 days decreases the median listeriosis cases in the elderly population from 228 to 197 (13.6%); and reducing home storage time to 10 days further decreases the cases to 154 (32.5%); (2) Eliminating storage above 8 °C or all storage times longer than 8 days, or combination of maximum 10 °C and maximum 11 days, led to a reduction in listeriosis cases of 50%; (3) Inclusion of a pre-retail lethal intervention in deli meats that produced a 1 log reduction in contamination at the start of retail would reduce the predicted deaths in the elderly population by nearly 50% (from 227 to 120 in the elderly population). Reducing contamination 2 logs would result in a 74% reduction.	ND	Medium: Different products considered	FDA-FSIS [17]
	Fermented meat	Growth (probability of growth, linear growth, GR at Tref), Survival (inactivation model as a function of lactic acid, salt and nitrate)	ND	ND	Medium: more complex predictive microbiology models	FAO-WHO [18]
	Hams	Growth (linear model, secondary model for GR as an empirical function of a_w_, storage temperature)	(1) During the first 6 days of storage at 10 °C, the expected increase in the listeriosis risk is 1 log; afterwards, the expected increase is around 1 log every 2 days; (2) If storage is at 4 °C, the risk increases in 1 log after 16 days; subsequent increases in 1 log would happen every week	ND	Low	Giovaninni et al. [19]
	Pre-packed deli meats/retail-sliced deli meats	Growth (linear model, sqrt model for GRs with and without inhibitors)	(1) The relative risk, on a per annum basis, of deli meats sliced at retail versus sliced in plants is 4.89; (2) In the elderly, use of GI in pre-packed deli meats reduces the mean annual deaths by 55%; (3) In the elderly, use of GI in retail-sliced deli meats reduces the mean annual deaths by 82%.	Regression tree analysis showed the most important determinants of risk are age of consumers, slicing location (i.e., retail or pre-packed) and presence of growth inhibitor.	Medium: The sensitivity analysis was carried out through the regression trees methodology; this model revised the FSIS-FDA (2003) model	Endrikat et al. [20]
	RTE meat and poultry deli meat	Growth (linear model, sqrt model for GRs with and without inhibitors)	(1) The formulation with GI in the elderly reduces annual death cases by 78%; (2) Retail-sliced deli meats present annual death cases 80% higher than those of pre-packaged deli meats; (3) An increase in the shelf-life from 10 to 40 days decreases the annual deaths by 13% (due to proper use of effective GIs that reduce deaths); (4) None of the simulated deaths are linked to the GI product.	Regression tree analysis showed the most important determinants of risk are age of consumers, slicing location (i.e., retail or pre-packed) and presence of growth inhibitor	Medium: The sensitivity analysis was carried out through regression	FSIS [21]
	Poultry and beef	Growth (linear model sqrt for growth), Inactivation (Bigelow model)	(1) Increasing mean storage temperature from 8 to 10 °C of meat stored for 3 days after cooking increases risk of listeriosis 62-fold for beef and 60-fold for poultry	ND	Low	Foerster et al. [22]
	Retail delicatessens	Growth (Yule’s model, secondary model for GR as a function of temperature, a_w_, nitrites, LAC and diacetate)	(1) When the the highly contaminated RTE food does not support the growth of LM, the predicted absolute risk increases two-fold in the susceptible population; (2) The risk from stores that have a highly contaminated RTE food that supports growth of LM is 6 times higher than the risk from stores that have a highly contaminated RTE food that does not support the growth of LM; (3) Most of the increase in the risk of products from highly contaminated stores results from cross-contamination to RTE foods that supports growth. (4) (i) Retail delis without niches and retail delis that control temperature produce lower listeriosis risk; and (ii) retail delis with incoming highly contaminated RTE foods (in particular, if they support growth), or retail delis with niches, produce higher listeriosis risk.	(1) When more LM cells enter the retail deli environment, the risk increases, regardless of whether these LM cells come from niches in the environment or from LM cells on incoming RTE food from the manufacturer; (2) High frequency of cross-contamination events (daily versus weekly) has greater impact than higher LM counts per cross-contamination event (100 versus 1000 CFU per contamination event).	High: Models cross-contamination in a deli grocery establishment by a discrete-event framework representing transfer from object to object, from food to object and from object to food	Pouillot et al. [23]
	Delicatessen meats/hotdogs	Growth (linear model, secondary model for EGR at 5 °C), Inactivation (Bigelow for death rate)	(1) The use of GIs led to a 110-fold reduction in the median cases of listeriosis for turkey deli meat, 78-fold for ham delicatessen meat, 56-fold for beef delicatessen meat and 49-fold for hotdogs; (2) Lowering the initial mean contamination levels from 75 CFU/g to 1 CFU/g resulted in corresponding 952-, 279-, 381- and 116-fold reductions, respectively.	**Output—listeriosis cases**: (1) consumer refrigerator temperature, (2) Consumer storage time, (3) EGR, (4) Retail storage temperature, (5) Temperature prior to retail (all the above with r = 0.30–0.43), (6) Lag time (−0.13 to −0.27)	Low: However, lag considers time elapsed between end processing and retail	Falk et al. [24]
	Packaged heat-treated meat products (cooked meat, sausage, pâté)	Growth (Baranyi with Jameson effect due to LAB, EGR5 secondary model and effect of lactate)	(1) Decreasing by 1–2 °C in the dynamic temperature profiles reduced cases of listeriosis per million servings by up to 37%; (2) Reducing maximum initial LM concentration by 2 log CFU/g decreases cases by 89%; (3) Decreasing time to consumption by 25% decreases cases by up to 38%; (4) Including lag time in the model reduces cases by 57%	ND	Medium: Time–temperature profiles from retail to consumption, and microbial competition models solved with 4th order Runge–Kutta algorithm	Pérez-Rodríguez et al. [25]
	Retail-sliced cooked meats	Growth (competition growth model LM- LAB, secondary model for GR as a function of pH, a_w_, nitrites)	(1) Setting a use-by date of 14 days from the time of slicing decreases the median annual listeriosis cases from 7 (no use-by date) to 0; (2) Reducing consumers’ storage temperature from a mean of 6 °C to 5 °C reduces the median listeriosis cases from 7 to 0.	ND	Low	Tsaloumi et al. [26]
	RTE cooked meat, RTE sausage, patê	Growth (Rosso model, EGR 5 °C)	(1) Across the 3 meat products, there is no strong difference in the probability of a product exceeding 100 CFU/g at the point of consumption between normal packaging (0.0672–0.0691) and reduced-oxygen packaging (0.0654–0.0678)	Risk is very sensitive to MPD. A shift in 0.5 log CFU/g can double the estimated risk. However, sensitivity analysis was conducted grouping various RTE food classes.	Low: Generic model; demands some knowledge in R software to utilise it	EFSA BIOHAZ [27]
	RTE cooked meat	Growth (Baranyi, Jameson competition growth model LM- LAB, secondary model for GR as a function of T, pH, a_w_, CO_2_, nitrites)	(1) Current cold-chain operating conditions; (2) Home refrigerator thermostat set at 4 °C; (3) Home refrigerator thermostat set at 7 °C; (4) Better thermal insulation of the refrigerator; (5) Lower air curtain flow rate in the display cabinet (50%); (6) Lower air curtain flow rate in the display cabinet (75%); (7) Higher air curtain flow rate in the display cabinet (125%); (8) Thermostat set in the display cabinet at 2.5 °C	Sensitivity analysis of the exposure model was carried out in Duret et al. [36]	High: the model, together with liste-riosis risk, assessed the energy consumption and the spoilage at time of consumption. DALYs and costs are used to express the results, and ranking of scenarios is carried out using multicriteria decision analysis	Duret et al. [28]
**Consumption**	Vacuum-packed and freshly sliced deli meats	Growth (linear model, secondary model for EGR)	(1) For initial LM at retail, levels lower than −2 log CFU/g did not affect mean mortality (death/serving); (2) After 10 days of storage at a mean temperature of 3 °C, there was no increase in risk (maximum risk reached) because the mMPD was attained; (3) Risk increased only when deli meats were kept for more than 18 h at ambient temperature; (4) Reducing maximum storage temperature appeared to be more effective at reducing risk than reducing refrigeration time for deli meats; (5) Initial contamination levels at retail had stronger impact on listeriosis risk than cross-contamination in the home.	**Output—mortality in intermediate age population**: LM level at retail (r = 0.29), Repeated use of leftovers (r = 0.17), EGR5 (r = 0.12), Refrigeration temperature (r = 0.09), Serving size (r = 0.09), Refrigeration time (r = 0.03)	Low	Yang et al. [29]
	Cooked chicken offal	0	ND	ND	Low	Kuan et al. [30]
	Cooked chicken offal	0	ND	ND	Low	Wai et al. [31]

GI: growth inhibitors; a_w_: water activity; LPD: lag phase duration; GR: growth rate; EGR_x_: exponential growth rate at x °C; LAB: lactic acid bacteria; LAC: lactic acid concentration; r: Pearson’s correlation coefficient; ND: not done; Bold: It is used to make a distinction for the grouping of the scopes of models.

## Data Availability

A BibTeX file containing clean records from the systematic review is available.

## Supplementary Materials

The following supporting information can be downloaded at: https://www.mdpi.com/article/10.3390/foods13030359/s1.
